# Editorial: Case reports in neuropharmacology 2022

**DOI:** 10.3389/fphar.2023.1181871

**Published:** 2023-06-06

**Authors:** Adrian Preda

**Affiliations:** Psychiatry and Human Behavior, University of California, Irvine, Irvine, CA, United States

**Keywords:** lamotrigine, dinalbuphine sebacate, CaMK2, propofol, pink urine syndrome, lymphadenopathy, valproic acid

The Research Topic of *Case Reports in Neuropharmacology 2022* represents the diverse range of research and clinical applications in this vast field. From the use of new medications for post-surgical pain management to the recognition of rare drug-induced side effects, the present Research Topic highlights the breadth and complexity of neuropharmacology research and practice. These case reports emphasize the importance of personalized approaches to medical treatment, careful monitoring of potential drug interactions and side effects, and continued research to improve patient outcomes in this field.

The four articles of this Research Topic share a common neuropharmacology background as well as an emphasis on patient outcomes. One of the highlights of this Research Topic is the variety of specific Research Topic covered, while at the same time each article highlights the importance of personalized approaches to medical treatment and the need for careful monitoring of potential side effects and drug interactions.

Liu et al. report on the use of dinalbuphine sebacate (DS), a mixed kappa opioid agonist/mu opioid antagonist, as part of a multimodal analgesia (MMA) protocol for morbidly obese patients undergoing laparoscopic sleeve gastrectomy. These patients are at an increased risk of opioid-related side effects, such as post-operative nausea and vomiting and respiratory depression. Obstructive sleep apnea, which is prevalent among patients with morbid obesity, further put these patients at risk for respiratory depression (Weingarten et al., 2015). In this context, a multimodal analgesia strategy which provide adequate perioperative analgesia at a low “opioid cost” could improve recovery in bariatric surgery. There are few reports on the use of dinalbuphine sebacate (DS), a non-controlled opioid medication with prolonged analgesic effects, good tolerability, and a non-addictive profile. In the case presented, a new MMA protocol incorporating DS for laparoscopic sleeve gastrectomy was utilized on a 46-year-old female with morbid obesity. The protocol consisted of an ultrasound-guided intramuscular DS injection, a transversus abdominis plane (TAP) block, and other analgesics. The patient achieved good perioperative analgesia, experienced an opioid-sparing effect, and displayed enhanced recovery with no pain for the following four months. This case report suggests that DS-incorporating MMA protocols is an efficacious and well tolerated intervention that could mitigate the risk of opioid-based analgesia protocol for morbidly obese patients undergoing laparoscopic bariatric surgery. By reducing opioid consumption and providing effective perioperative analgesia, DS may lead to improved patient outcomes, fewer opioid-related side effects, and enhanced recovery. Further research, including randomized controlled trials, is warranted to validate the efficacy and safety of DS in MMA protocols for bariatric surgery and other surgical procedures.

Zhang et al. describe a rare side effect of propofol anesthesia known as pink urine syndrome (PUS) in a non-obese patient following thoracoscopic wedge resection of pulmonary nodules. This report adds valuable information to the literature on PUS, specifically regarding its occurrence in non-obese patients and the potential role of propofol anesthesia in its development. PUS has been previously associated with urinary uric acid (UA) disorders, reported in morbidly obese patients undergoing gastric bypass surgery and/or propofol anesthesia in individuals with preexisting UA metabolic disorders (Deitel et al., 1984; Tucker and Perazella, 2019). However, the incidence of PUS in non-obese patients after exposure to propofol is infrequent, and there is limited literature on long-term follow-up after PUS. This report adds valuable information to the literature on PUS, specifically regarding its occurrence in non-obese individuals and the potential role of propofol anesthesia in its development. This case report is an important reminder that not all sudden and spectacular changes automatically carry a risk for a negative outcome.

Dwyer et al. report on a newly recognized neurodevelopmental disorder caused by mutations in genes encoding calcium/calmodulin-dependent protein kinase II (CAMK2) isoforms. The article discusses the impact of different medications on CAMK2 activity and associated calcium signaling and suggests personalized treatment regimens based on CAMK2 catalytic activity. The hypothetical treatment framework proposed by the authors is an important step towards clarifying the questions that will guide further research. This is an essential consideration, as dysregulation of calcium signaling can have profound consequences for neuronal development, function, and survival (Berridge et al., 2000). The report represents an important advance in our understanding of a understudied neurodevelopmental disorder associated with CAMK2 mutations. The authors’ proposed personalized treatment regimens, based on CAMK2 catalytic activity, offer a promising avenue for further research and potential therapeutic development. As our knowledge of CAMK2 and its role in neurodevelopment continues to expand, it is hoped that targeted interventions can be developed to improve the lives of those affected by this complex disorder. 

Duan et al. discuss a rare case of severe skin rash and lymphadenopathy associated with the use of lamotrigine and valproic acid in a patient with bipolar disorder type I. The case highlights the potential for severe skin reactions and lymphadenopathy associated with the use of these medications and emphasizes the need for caution during titration and early withdrawal of both when signs of hypersensitivity appear. While the dermatologic toxicity of lamotrigine is known, this report is a reminder of the importance of careful consideration of the unknown variables that can complicate the course of apparently well charted adverse effects risk trajectories.

Together, these articles underscore the importance of personalized approaches to medical treatment, particularly in patient populations with specific vulnerabilities or susceptibilities to certain side effects. They also highlight the need for careful monitoring of potential drug interactions and side effects to ensure the best possible patient outcomes. By understanding the neuropharmacology underlying different medical conditions and treatments, we can continue to develop effective and personalized approaches to medical care.
